# Will Future Measurement Needs of the Semiconductor Industry Be Met?

**DOI:** 10.6028/jres.112.002

**Published:** 2007-02-01

**Authors:** Herbert S. Bennett

**Affiliations:** National Institute of Standards and Technology, Gaithersburg, MD 20899

**Keywords:** economic forces, measurement needs, metrology, semiconductor industry, standards, technological innovation, technology roadmaps, United States Measurement System (USMS)

## Abstract

We discuss the ability of the nation’s measurement system to meet future metrology needs of the semiconductor industry. Lacking an acceptable metric for assessing the health of metrology for the semiconductor industry, we identify a limited set of unmet measurement needs. Assuming that this set of needs may serve as proxy for the galaxy of semiconductor measurement needs, we examine it from the perspective of what will be required to continue the semiconductor industry’s powerful impact in the world’s macro-economy and maintain its exceptional record of numerous technological innovations. This paper concludes with suggestions about ways to strengthen the measurement system for the semiconductor industry.

## 1. Introduction

As the United States’ national measurement institute, the National Institute of Standards and Technology (NIST) assists all stakeholders in selected fields with their measurement and standards needs. The goal of NIST’s involvement is to enhance efficiency and productivity and increase the rate of technological innovation. NIST is not a regulatory agency, but rather serves as a neutral third party, often providing technical input in matters related to measurements and standards to a variety of customers: industry, universities, national and international standards committees, where appropriate. NIST recently accepted the challenge to see whether the United States Measurement System (USMS) is meeting the nation’s measurement needs, and thus produced an assessment of the USMS in January 2007 [1]. The semiconductor industry was one of the areas considered in terms of its measurement needs for accelerating technological innovation. The set of measurement needs required to meet the technical challenges cited in the Metrology Chapter of the 2005 International Technology Roadmap for Semiconductors [2] was used to examine the status of measurements and standards for accelerated technological innovation in the semiconductor industry. This paper presents an analysis based on selected case studies of those measurement needs that the semiconductor industry submitted to NIST.

The successes of the semiconductor industry led to major impacts on many other industries, such as those that support computers, networks, information technologies, entertainment, healthcare, and defense. Advances powered by semiconductors gave businesses and consumers new flexibility, freedom, and opportunity. Activities that once confined people to the home or office can now be performed at any time, any place, and almost anywhere in the world. The semiconductor industry is bringing new opportunities, socio-economic advances, and progress to nations and societies around the world. The semiconductor industry contributes substantially to overall global economic health and is entering an era of global private-public-government partnerships to meet future challenges.

The scope of the analysis given here is primarily from the perspective of the 2005 Edition of the International Technology Roadmap for Semiconductors (ITRS) [2], which includes semiconductor memories, microprocessors, signal processors, radio frequency and analog/mixed signal circuits, logic devices, and emerging research devices and materials. It does not include semiconductors for power electronics used in transportation systems and for optoelectronic components used in telecommunications systems.

In this paper, we are analyzing the USMS as a system that supports the semiconductor industry. Using a limited data base, we have taken a “snapshot”, not a “video”, of the semiconductor part of the USMS. We are not assessing measurement needs in detail; nor any area in depth. Neither are we assigning any priorities for addressing the measurement needs and barriers to technological innovation in the semiconductor industry. Considering these caveats; we certainly do not suggest that readers limit their horizons to the focus of this analysis on specific topics cited in Appendix 1. Also, the illustrative topics given here may not lead in the future to optimal solutions and, therefore, may not be the actual solutions adopted by the semiconductor industry. For example, optical scatterometry is not cited in Appendix 1, but it has great potential for the difficult challenge cited in Table 1 of nondestructive, production worthy wafer inspection for critical dimensions.

## 2. Semiconductor Industry Structure

The semiconductor industry consists of companies, trade associations, research and development consortia, universities, and governments that contribute to the economic well-being of many nations. Its main products and players are listed in the following two sub-sections.

### 2.1 Semiconductor Products

The International Electronics Manufacturing Initiative (iNEMI), http://www.inemi.org, and the Semiconductor Industry Association (SIA), http://www.sia-online.org, have essentially orthogonal ways to classify semiconductor products. The iNEMI considers broad applications and their technical attributes, which it calls product emulators. The SIA considers semiconductor components, each of which has many applications in the iNEMI classification scheme.

The 2004 iNEMI Roadmap [3] uses the following seven product applications and technology attributes as the framework in which to present its findings:

Portable / Consumer - High volume consumer products for which cost is the primary driver.
System in a Package - Complete function provided in a package to system manufacturers.
Office Systems / Large Business Systems - Products which seek maximum performance from a few thousand dollar cost limit to almost no cost limit.
Network / Datacom / Telecom Products - Products that serve the networking, datacom and telecom markets and cover a wide range of cost and performance targets.
Medical Products - Products which must operate within a high reliability environment.
Automotive - Products which must operate in an automotive environment.
Defense and Aerospace - Products which must operate in extreme environments.

The SIA [4] presents market data and trends for the following products: Discrete Components, Optoelectronics, Analog, Metal Oxide Semiconductor (MOS), Logic, Microprocessor, Microcontrollers, Digital Signal Processors, Dynamic Random Access Memory (DRAM), and Flash Memory.

### 2.2 Major Players

The major players are: Device Manufacturers, Equipment Manufacturers, Materials Manufacturers, Sub-Systems and Components Parts Suppliers, Factory Control and Facilities Management, Providers of Software for Design and Manufacturing, Manufacturing Services Providers, Business Services and Consulting Providers, Vendors for Support Products and Consumable Materials, Trade Associations, Research and Development Consortia, Universities, Governments, World Trade Organization, Regulatory Agencies, and Legislators.

The ITRS has more than 1200 international contributors. Their affiliations are chip makers 58 %; equipment and materials suppliers 21 %; consortia, research institutes, and universities 18 %, and others 3 %. Their geographic locations are USA 52 %, Japan 18 %, Taiwan 16 %, Europe 9 %, and Korea 5 %. The websites: http://www.inemi.org/cms/about/members.html, http://www.sia-online.org/mem_list.cfm, http://wps2a.semi.org/wps/portal/_pagr/119/_pa.119/214, and http://www.sematech.org/, contain lists of members, respectively, for iNEMI, SIA, SEMI, and SEMATECH/International SEMATECH.

Several semiconductor trade associations formed the World Semiconductor Council (WSC, http://www.semiconductorcouncil.org) in 1999. The WSC consists of the following trade associations from around the world: European Semiconductor Industry Association (EECA-ESIA, http://www.eeca.org), Japan Electronics and Information Technology Industries Association Semiconductor Board - Japanese Semiconductor Industry Association (JEITA-JSIA, http://semicon.jeita.or.jp/en/), Korea Semiconductor Industry Association (KSIA, http://www.ksia.or.kr), U.S. Semiconductor Industry Association (SIA, http://www.sia-online.org), and Taiwan Semiconductor Industry Association (TSIA, http://www.tsia.org.tw). The purpose of the WSC is to promote cooperative semiconductor industry activities, and expand international cooperation in the semiconductor sector in order to facilitate the healthy growth of the industry from a long-term, global perspective.

Other major players include research consortia. These are often industry-university-government partnerships. Examples include the Semiconductor Research Corporation (SRC), http://www.src.org/member/about/membercompanies.asp; Microelectronics Advanced Research Corporation (MARCO)/Focus Center Research Program (FCRP), http://fcrp.src.org/Default.asp?bhcp=1; Advanced Materials Research Center, http://www.amrctx.org/; Interuniversity Microelectronics Center (IMEC), http://www.imec.be; and Semiconductor Leading Edge Technologies, Inc. (SELETE), http://www.selete.co.jp/index_e.html.

## 3. Economic Dimensions of Industry

The semiconductor industry greatly affects global economic growth. Just as the industry’s strength provides a leading indicator of the world’s economic health, advanced semiconductor products and systems contribute substantially to new opportunities, growth, and development in nations around the globe. The semiconductor industry enriches the lives of people the world over, by improving health and safety, enhancing education and learning, and offering new opportunities for work, recreation, and entertainment. Free and open international trade is a primary engine of global growth and development. The semiconductor industry has enabled, and continues to enable and sustain many other economic sectors that contribute significantly to the gross world product (GWP).

### 3.1 Trends in the Semiconductor Industry

The following trends in the semiconductor industry are occurring concurrently, with rapid changes in applications of semiconductors:

- The competitiveness among many semiconductor manufacturers is shifting from an emphasis on processing technologies for fabrication, to a much greater emphasis on product design, architecture, algorithm, software, and life-cycle evolution.
- Increased costs for Research and Development and production facilities are becoming significant.
- Process technology life cycles are becoming shorter.
- There is an emphasis on faster characterization of manufacturing processes, assisted by increased modeling and simulation for nanotechnologies to offset the greater increase in costs associated with measurements.
- Demands for more and more bandwidth due to the digitization of everything and the need to decrease latency.

**Fig. 1:**
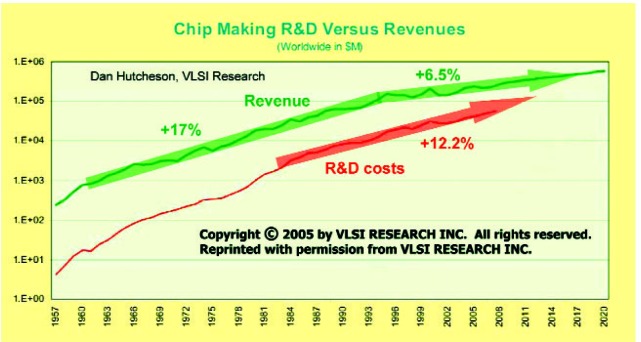
Research and development for semiconductor chip manufacturing compared to revenues.

Figure 1 compares R&D expenses and revenues for semiconductor chip manufacturing. From 1966 to 1996, the annual growth rates for expenses and revenues were comparable and averaged about 17 % during those 3 decades; but the annual growth rates from 2004 to 2020 for “R&D expenses” are expected to be 12.2 %, whereas the annual growth rates from 2004 to 2020 for “revenues” are expected to be only 6.5 %. This trend of today’s semiconductor industry is not sustainable if the semiconductor industry is to continue its decades of being a powerful deflationary force in the world’s macro-economy.

### 3.2 Size

According to the 2005 SIA Annual Report, more transistors were produced last year, and at a lower cost than grains of rice [5]. The worldwide transistor production exceeds 10^18^ transistors per year. Each transistor costs less than 100 nano-dollars and has dimensions less than 100 nm. These three numbers illustrate the effects of *Moore’s Law* [2] — a historical observation by Intel executive, Gordon Moore, found that the functionality per chip (bits and transistors), doubles every 1.5 to 2 years. Constantly deploying new technology innovations drives exponential increases in the number of transistors per chip, and simultaneously reduces the cost per function. The result is that semiconductor chips become faster, better, and cheaper every year [6]. The majority of the world’s semiconductor manufacturing capacity, 75 %, lies outside the United States [7]. The U.S. industry share of the world’s semiconductor manufacturing capacity declined from about 28 % in 1999, to less than 25 % in 2005. A steeper decline in leading-edge semiconductor manufacturing capacity—from about 36 % in 1999, to about 14 % in 2005—also occurred. These two declines in the U.S. share accompanied a decline in the U.S. share of world expenditures for semiconductor research, development, and manufacturing capacity—from a high of 45 % in 1998, to less than 30 % in 2005. These declines are significant for an industry that is capital-rather than labor-intensive.

### 3.3 Recent Revenues and Growth

The Semiconductor Industry Association (SIA), http://www.sia-online.org, released, on 16 November 2005, its annual forecast of global semiconductor sales. It projects a compound annual growth rate of nearly 10 % for the forecast period of 2005 through 2008. This forecast projects that worldwide sales of microchips will reach $309 B in 2008. This is an increase of 45 % from the $213 B record level of 2004. The forecast calls for 2005 sales to increase by 6.8 %, to $227.6 B, followed by increases of 7.9 %, to $245.5 B in 2006; 10.5 %, to $271.3 B in 2007; and 13.9 %, to $309.2 B in 2008. This forecast also contains the distributions for the same period among the geographic markets of the Americas, from $39.1 B to $51.1 B, or a 31 % increase; Japan, from $45.5 B to $56.7 B, or a 24 % increase; Europe, from $39.4 B to $51.0 B, or a 29 % increase; and Asia-Pacific from $88.8 B to $150.4 B, or a 69 % increase [8]. However, in June 2006, the SIA raised its forecast for 2006 worldwide sales growth from the above 7.9 % to 9.8 % [9].

Consumer products now account for over half of the demand for semiconductors [10]. For example, third generation (3G) cellular phones now have a much higher semiconductor content than they did a couple of years ago. Also, they now represent 50 % of the cellular phone market, compared to only 5 % a few years ago.

### 3.4 Impact on Global Economy

The “general purpose nature” of semiconductor technology has widespread impact on many other industries, because its considerable productivity growth means the same performance level for substantially less cost from one year to the next. The economic value of Moore’s Law has been its powerful deflationary force in the world’s macro-economy. Inflation is a measure of price increases, without any qualitative change in performance. So, when the price per function is declining, it is deflationary. This long-term deflationary effect of semiconductors has never been fully accounted for in statistics and economics. For example, the decline in price per bit has been stunning. In 1954, five years before the integrated circuit was invented, the average selling price of a transistor was $5.52. Fifty years later, in 2004, the price had dropped to a billionth of a dollar. A year later, in 2005, the cost per bit of dynamic random access memory (DRAM) is an astounding one nanodollar (one billionth of a dollar). Applying this impact of the semiconductor industry’s successes and growth to other areas leads to statements like:

1. “In 1978, a commercial flight between New York and Paris cost $900 and took seven hours. If the principles of Moore’s Law were applied to the airline industry, that flight would now cost about a penny and take less than one second”[11].
2. “If the automobile industry advanced as rapidly as the semiconductor industry, a Rolls Royce would now get half a million miles per gallon, and it would be cheaper to throw it away than to park it”[12].
3. “If the automobile had advanced in the same way as the semiconductor industry over the last 25 years, a Rolls Royce today would still cost about US$ 320,000, but would have about four million tires and carry about five million passengers, all of whom would be required to be very small”[13].

Collectively, these three statements illustrate that the Moore’s Law type of progress is not appropriate, and therefore, does not occur, for many industries [12].

### 3.5 Business Challenges and Drivers

The great successes of the semiconductor industry required decades of high-risk investments. Its major business challenges are to continuously create new knowledge and develop it into technologies that drive the global economy, guarantee security, and improve health and quality of life. Stronger public-private-government partnerships will be needed to support R&D for technological innovations so that the deflationary attributes of past semiconductor products continue with benefits for all.

Some semiconductor products are becoming similar to commodities for which success in technological innovation is necessary, but not sufficient for market success. This introduces new business models as the industry completes a major re-structuring, by transferring its center of manufacturing competence from the original equipment manufacturers (OEM) to electronics manufacturing services (EMS) providers and original design manufacturers (ODM). These new business models also must account for the movement of manufacturing and manufacturing support to China, from North America, Europe, and other Asian countries [14].

Additional business challenges include regulatory and legislation issues. The two European Union directives on restricting use of certain hazardous substances (RoHS) and on managing waste from electrical and electronic equipment (WEEE), now govern the material content and end-of-life management of semiconductor and other electronic products. Such environmental legislation affects the design and recycling of products worldwide and requires the industry to share detailed material content data of their products and components. Manufacturers must remove environmental “Materials of Concern” such as lead to meet these regional requirements. Measurement issues and ambiguities in some of the regulations and legislation exist, and contribute to investment risks. The semiconductor industry has many concerns about its impact on the environment, health, and safety, particularly, about its emissions, water use, and power use in the context of global warming [15].

Other drivers are markets and the semiconductor technologies themselves. Basic computing, communications, and entertainment products are merging, and their combined performance requirements drive increases in product functionality. The approaching end of traditional semiconductor scaling has its own consequences that include:

- The gradual but certain reduction in an emphasis on microprocessor frequency as a performance metric; and
- The corresponding increase in importance of the system’s data throughput or bit rate as a performance metric.

Non-technical challenges and drivers that affect technological innovation include:

- Effective protection of intellectual property;
- Adherence to international standards as much as possible;
- International rules and domestic regulations that are consistent with each other, and with open and competitive markets that are fair to all;
- Legislation and regulations that i) are nondiscriminatory, ii) based on sound and widely accepted scientific principles, iii) based on publicly available technical and medical information, and iv) do not impede the effective functioning of the semiconductor market;
- Scientific and engineering workforce.

## 4. Technology, Metrology, and Technological Innovation

In 2000, the semiconductor industry entered the nanotechnology era by shipping products with nanoscale horizontal features (e.g., gate lengths less than 100 nm) and with gate oxide thicknesses close to 1 nm. Early in 2004, the industry implemented the 90 nm node in volume production that has a physical gate length less than 40 nm for some applications. This reinforces the industry’s position as a true nanotechnology pioneer, through continued technology advances at the pace of Moore’s Law.

Innovative methods are needed to improve cooling and reduce operating junction temperatures—due to large leakage currents and increases in chip power, especially increased power per unit area. These shifts in metrics to assess system performance will generate an increased demand for higher bandwidth to and from the microprocessor, memory, and other components; more accurate and precise measurements at higher data rates and higher RF frequencies; and for temperature measurements at high spatial resolutions. Optical systems may provide part of the solution for thermal management, particularly if optical integrated circuits become available for high-volume applications [16].

### 4.1. Essential Technologies and Measurements

Scientists and engineers believe that advances in semiconductor technology can continue to progress, according to Moore’s Law, for another 10 to 15 years. However, there are physical, technological, and economic limits to continued scaling of semiconductor components using today’s main-stream complementary metal oxide semiconductor (CMOS) technology. Scientists generally agree that these limits will be reached around 2020. Without new breakthroughs and new technological innovations for metrology, the rate of progress for the semiconductor industry will slow considerably between now and 2020. This, in turn, will slow the rate of progress for all the related technologies such as information technology and communication systems that depend on semiconductors. But, the limits of current CMOS-based technologies do not necessarily mean an end to progress. With sustained and coordinated commitment to basic research and deploying research results, the semiconductor industry, supported by academia and the world’s governments, should be able to have resources such that the technological barriers are overcome, and progress continues as in the past. The nanotechnology era will require new materials, new device structures, and new manufacturing methods; each of which will demand new measurement techniques. The challenges are enormous, but so are the rewards for success. As Gordon Moore commented on his pervasive law a few years ago, “No exponential lasts forever; but, forever can be postponed.” The global semiconductor industry must invest heavily to postpone it.

### 4.2 Importance of Technological Innovation

For four decades, the semiconductor industry has maintained a rapid pace of technological innovation based on its ability to invest in advanced measurements to support each succeeding technology generation. Many of the innovations resulted principally from its ability to decrease, exponentially, the minimum feature sizes used to fabricate semiconductor-integrated circuits. The most significant trend is the decreasing cost-per-function, which has led to significant improvements of productivity and quality of life through proliferation of computers, electronic communication, and consumer electronics.

New technological innovations and advanced processing of semiconductor devices and circuits require measurements for verifying critical dimensions, microscopy, lithography, front-end processing, interconnect performance, low-dielectric constant materials used with copper, high-dielectric constant materials for insulators, materials and contamination characterization, and emerging research devices and materials.

### 4.3 Challenges and Barriers to Enhancing Performance

#### 4.3.1 Near-Term (2006 to 2013) Grand Challenges

- Extreme ultra violet lithography is proposed as a successor to argon fluoride (ArF) lithography. Immersion technology has the potential to extend optical lithography down to 32 nm half-pitch.
- Control of critical dimensions has become one of the most difficult issues in lithography and etching as a result of the aggressive scaling of gate lengths.
- Computer simulations and modeling of front-end processes for nanometer structures are key challenges for the prediction of device performance and for decreasing the time to develop technological innovations; design and simulation tools are main roadblocks to more rapid introduction of new technologies [17].
- Signal isolation, especially between the digital and analog regions of the chip, is a particular challenge for scaled technologies and increased integration complexity.

#### 4.3.2 Long-Term (2014 and beyond) Grand Challenges

- Fundamental issues of statistical fluctuations and process variations for sub-15 nm gate length devices and the impacts of quantum effects, line edge roughness (LER), and line width roughness (LWR) are not understood well and limit deploying new measurement techniques and the pace of technological innovations.
- The resolution and precision measurements for critical dimensions (CD) down to 7 nm and LWR metrology of 0.8 nm in 3 standard deviations (σ) with the required overlay accuracy of 2.8 nm in 3σ or better in 2019 is extremely challenging. Without metrology and inspection tools having sufficient accuracy and resolution, CD control improvements and process control will be difficult to achieve.
- Non-destructive measurements that do not charge or contaminate the surface and high-resolution wafer and mask level microscopy for measuring the critical dimensions of 3-dimensional nanostructures and defect detection are required.

### 4.4 Measurement Challenges and Barriers to Technological Innovation

The rapid introduction of new materials, processes, and 3-dimensional structures places great demands on metrology. The time between when new measurement techniques become available for manufacturing and when high-volume production begins has decreased substantially. In the past, measurement techniques were two or more generations ahead of the technology being used for production. But today, that is not the case. In some cases, the lack of measurement techniques inhibits progress.

Extrapolating from a 1998 NIST study of the semiconductor industry suggests that the costs of measurements performed during semiconductor manufacturing today is about $9 B. This amount is probably a lower limit because the extrapolation assumed implicitly 1998 lithography costs, not recent lithography costs [18]. Lithography costs and those of measurements to support lithography tend to increase with each succeeding technology generation.

Table 1 lists short- and long-term difficult challenges and issues from the 2005 ITRS perspective. Appendix 1 contains the shorthand notation for relating semiconductor case studies of measurement needs, in Appendix B of reference 1, to specific challenges and issues in the 2005 ITRS. Table 1 represents the consensus of more than 50 experts from around the world who are members of the Metrology ITRS Technical Working Group (ITWG). These are the metrology challenges and issues that must be addressed if the semiconductor industry is to continue its historic successes in deploying technological innovations that result in decreasing the cost per function and the volume of material per function (i.e., increasing the number of functions per unit volume) with each new technology generation [19].

## 5. Measurement Needs (MNs)

The 2005 ITRS presents technology requirements for CMOS ICs and post-CMOS ICs used in memories, microprocessors, digital signal processors, logic, networks, wireless communications, and other computing products. These products constitute over 75 % of the world’s semiconductor consumption. However, the 2005 ITRS does not emphasize measurement needs to support new technology innovations for other high volume applications of semiconductors such as optoelectronics (light emitting diodes, lasers, digital video, digital versatile disk (DVD) players, displays, optical communications, and the like) and power electronics (hybrid autos and trucks, all electric vehicles, other transportation systems, power distribution, and the like).

Because research and development responsibilities are shifting from original equipment manufacturers (OEM) to equipment and materials suppliers (EMS), the international semiconductor industry should formulate new ways, with academia, governments, and consortia, to meet its measurement needs; a few of which are cited in Appendix 1 of this analysis. New ways, also, are needed to deploy emerging technologies and innovative measurement techniques in the manufacturing process. These new ways of working together globally will have to be consistent with viable business models that are required: 1) to maintain decreasing the cost per function; 2) to maintain increasing the number of functions per unit volume; and 3) most importantly, to continue the positive deflationary effects the semiconductor industry has on many other economic sectors around the world.

### 5.1 Semiconductor Measurement Needs

Appendix B of reference 1 is a compilation of over 330 case studies of measurement needs for many areas. Semiconductor industry submitted 14 case studies for the area of semiconductors that are listed by title in Appendix 1. These 14 case studies are based on the 2005 ITRS Metrology Chapter [20]. They are the limited set of measurement needs that represent or serve as a proxy for the galaxy of measurement needs challenging the semiconductor industry, and address the major challenges for technological innovations in:

- factory controls,
- detecting nanoscale particles,
- 3D processing with smooth sidewalls,
- cluster-tools for compound semiconductors,
- RF isolation,
- test structures to verify processes and performance,
- characterizing interfacial layers,
- 3D distributions of dopants, defects, and atomic concentrations, and
- replacements for conventional active and passive devices such as 3D nanostructures and spintronics.

The majority of these semiconductor measurement needs are at the applied R&D stage of technology innovation. This stage concerns novel research and new findings for which conventional wisdom suggests that regulations are probably not very relevant. However, both domestic and foreign regulations may affect all stages of technological innovation, from basic research to end-use. During the applied R&D stage, regulations historically have neither limited nor enhanced the availability or accessibility of technology innovation. But, this historical pattern may be changing for nanoscale materials and devices. During the production, market, and end-use stages, regulations may have a critical role in technology development and deployment. Continually evaluating whether regulations will inhibit or enhance technological innovation and its successful deployment is particularly important for the health of the USMS.

The 14 case studies of measurement needs listed in Appendix 1 address the difficult challenges and issues in Table 1 [21]. Each case study of measurement needs appears in an element of the Summary of Issues column of Table 1, for which the need is a response to the challenge and issue discussed in that row of Table 1. Appendix 1 gives the shorthand notation for identifying each case study in Table 1 and locating the case study in Appendix B of reference 1. Some measurement needs appear in more than one element of Table 1. Placing the measurement needs in the elements of Table 1 highlights their roles in addressing specific difficult challenges and issues.

### 5.2 Common Attributes Among Case Studies of Measurement Needs

Because of the continued decrease in the cost-per-function, and increase in the number of functions-per-unit volume with each deployment of a new technology generation during the last 40 years, the contributions of the USMS to the international measurement system in support of the global semiconductor industry were adequate.

However, the existence of so many measurement needs, a few of which are listed in Appendix 1, indicates that the global semiconductor industry now must manage ever increasing risks associated with technological innovations to go beyond fully-scaled CMOS and develop the next switch.

One common attribute among the 14 case studies of semiconductor measurement needs presented in Appendix B [1] is that no one region, country, or company has the R&D resources to provide the solutions on its own. International collaborations, consortia, and partnerships will most likely be needed to provide solutions. This is especially true in the case of the lithography measurement needs both for fully-scaled CMOS and beyond CMOS. To date, all proposed-candidate technologies for going beyond CMOS, which might be used for general purpose computing and networking applications, still require very expensive lithography of some sort and do not offer clear paths to high-volume, reliable manufacture. The few candidate technologies, based on self-assembly, appear to be limited to specific applications such as processing images. These candidate technologies also do not offer clear paths on how to make connections to mature, fully scaled CMOS technologies.

Another common attribute of these needs is that meeting them requires improved fundamental understanding of chemistry, materials, and condensed matter physics over several lengths of scale, from microscopic to atomic dimensions. As devices shrink in size to nanometers, performing measurements on them becomes more costly and time-consuming. This means that computer simulations are now very critical for advances in semiconductors and other nano-technologies. Discussions on modeling and simulations for nano-materials and nano-technologies appear in such documents as the chemical industry’s roadmap http://www.chemicalvision2020.org/nanomaterialsroadmap.html.

Other common attributes include:

- Decreasing the time to perform measurements, especially those used in manufacturing.
- Developing new measurement technologies and deploying new measurement instrumentation.

But, none of the 14 case studies addressed the impact of international and domestic regulations on innovation in the semiconductor industry. For example, consider the impact that is discussed in http://www.itrs.net/Links/2005ITRS/ESH2005.pdf.

Since 1999, several trends have appeared that suggest the contributions of the USMS in support of the future semiconductor industry may not continue to be adequate unless substantial infrastructural changes occur. These trends include:

- The globalization of many organizations, beginning with the National Technology Roadmap for Semiconductors, becoming the ITRS in 1999. The ITRS was followed by others, such as NEMI becoming iNEMI, SEMATECH adding the subsidiary International SEMATECH, and SEMI changing the “I” from Institute to International.
- As device complexity increases, with higher densities of devices to lower cost per function, the associated R&D costs increase considerably. Semiconductor R&D costs are increasing at a rate that is not sustainable by any one company [22].
- The gap between R&D funds needed to meet ITRS goals, many of which are measurement needs, and available R&D funds is increasing. The SRC estimates that in 2003 the effective research gap for the U.S. based producers was $1.5 billion, when the research gap was adjusted for redundant research and the presumed unavailability of some offshore data [23].
- The investment risks are increasing, not only due to the technologies themselves, but to regulations and legislation as mentioned in the last paragraph of Section 3.5. These risks make international collaborative R&D more attractive as a way to reduce costs. Costs of development, costs of ownership, and risks are too high for any one region to afford.
- Many technical challenges are more difficult than originally thought, and there is scant time to overcome them before introducing the next technology generation, if the cost per function is to continue to decrease at its historical rate.
- Declining percentages of U.S. Institute of Electrical and Electronics Engineers (IEEE) membership and U.S. authorship of papers in the IEEE Transactions on Electron Devices (T-ED). The percentages for Asia and Europe are increasing, while those for the U.S. are decreasing. The Asian percentages are increasing, typically by a factor of 2 more than the European percentages. By 2014, the IEEE forecasts that less than 50 % of its members will be from the U.S. [24]. During 2004, only 70,000 engineers graduated in engineering in the U.S., whereas 600,000 engineers graduated in China and 350,000 graduated in India [25].
- Anticipated U.S. shortfall of engineers needed for semiconductor and nanotechnology jobs in the foreseeable future. The number of students enrolling in EE and CS majors is also starting to drop. There was a 1 % to 5 % decline in enrollments in 2004, compared to 2003, and many schools are reporting that interest level in these majors is declining [26].
- Recently, five independent studies concluded that the U.S. is at risk of losing its leadership in technology and innovation, with consequences for our future economic prosperity and national security. As each study was completed, the chorus became louder and urged policy makers to take significant steps to address this problem; a potential technological tsunami. The SIA website has two-page digests of the findings and recommendations for each of the five studies [27]:
  - The National Academies’ “The Gathering Storm” Report
  - The President’s Council of Advisors on Science and Technology’s (PCAST’s) “Sustaining Innovation Ecosystems” Report
  - The National Innovation Initiative’s “Innovate America” Report
  - The Defense Science Board’s “High Performance Microchip Supply” Report
  - “Tapping America’s Potential: The Education for Innovation Initiative”

As a result of these studies and others, Congress has proposed legislation to encourage increased innovation.

Assuming that the above trends will continue and assuming scientific and engineering prowess is critical for addressing the measurement needs of the semiconductor industry and technological innovation in the marketplace, we then may conclude that more and more USMS solution providers will have to come from foreign countries, especially Asian countries. This shift in the geographic location of USMS solution providers may become a necessary option or compromise for maintaining the health of the portion of the USMS that supports the global semiconductor industry.

### 5.3 Déja Vu

Considering the future and role of the USMS in the international semiconductor industry, we should remember Prof. Derek D. de Solla Price’s earlier results in the context of the semiconductor industry’s measurement needs today. Prof. Price summarized, in 1971 and 1963, his research based on market analyses, interviews of leading technologists, and examinations of patents, archival publications, and citations for patents and publications [28]. Based on his research data, Prof. Price formulated relationships, then, that are today relevant to the mainstream portion of the semiconductor industry, which is the focus of this analysis. Commercial technologies and markets have two major phases of development; growth and saturation.

During the growth phase, he discerned such relationships as:

- High quality technology development grows at a slower rate than low quality technology development.
- The number of high quality developments is proportional to the *n*th root of the total number of developments where *n* is greater than 2 or 3 and depends on the technical field.
- Resources and funds devoted to a given technology are proportional to the *n*th power of the number of people working on that technology. This implies technology growth will be limited, eventually, by a lack of resources, both financial and human.
- Doubling the size of a technological effort does not double the metric for useful results. His data suggests that the useful results vary as the *m*th root of the size of the effort, where *m* is between 2 and 4 for most technologies.

The factors that determine the saturation phase include:

- The finite extent of any economy means that the required resources to continue the advancement of a given technology, such as semiconductors, cannot exceed the available money supply.
- The finite number of workers means that one technology, such as semiconductors, cannot capture most of the human resources to advance that technology.

According to Prof. Price, technology deployment is more like the arts and may be localized as language is localized. For the semiconductor industry, this last statement becomes equivalent to the statement that the ITRS gives technology requirements and measurement needs. It remains for local decision-makers to select those requirements and needs for which local resources will be used to provide solutions, e.g., solutions to the 14 measurement needs listed in Appendix 1. This is just what many of the consortia listed above are doing. They select which challenges to accept.

### 5.4 Strengthening the Semiconductor Industry

#### 5.4.1 Computers, Networks, and Visualization

As the percentage of U.S. resources devoted to technological innovations for the semiconductor industry decreases, relative to the percentages of other countries and regions, the U.S. must find ways to offset the negative effects that this percentage decrease may have on its leadership in semiconductor technologies. One way would be to excel at computing, networking, and visualization techniques to understand advanced materials processing, design, and bring innovative semiconductor products to market quicker and cheaper. According to the IDC’s July 2004 report [29] about the return on investment in modeling and simulation for materials science, increasing resources for modeling and simulation that support experiments lowers the overall costs for experimental research and development and leads to net savings. This report presents data that suggest a return on investment (ROI) of $3 to $9, for every dollar spent on modeling and simulation by:

- Increasing the productivity of expensive measurements, and
- Lowering the cost per R&D deliverable or outcome through computer simulations: to guide and design the experiments, control the experiments during data acquisition, and interpret more rigorously with immersive visualization methods the data from which useful information is extracted.

#### 5.4.2 International Consortium of National Measurement Institutes

Another way to offset the anticipated decrease in the U.S. share of R&D for semiconductor metrology innovation would be the formation of international consortia composed of members from National Measurement Institutes (NMIs) for which meeting the measurement needs cited in Appendix 1 is also critical to their respective economies. Candidate NMIs with which NIST could collaborate are: Institut Belge de Normalisation/Belgisch Instituut voor Normalisatie (IBN/BIN), Belgium; Bureau National de Metrologie (BNM-INM), France; Physikalisch-Technische Bundesanstalt (PTB), Germany; National Metrology Institute of Japan (NMIJ) and National Institute of Advanced Industrial Science and Technology (AIST), Japan; Korea Research Institute of Standards and Science (KRISS); Center for Measurement Standards, Taiwan; National Institute of Metrology (NIM), China; National Physical Laboratory (NPL), United Kingdom; and NMi Van Swinden Laboratorium B.V., The Netherlands.

#### 5.4.3 Industry, University, and Government Consortia/Teams

The measurement needs (MN) and barriers listed in Appendix 1 and cross-referenced to Appendix B of reference 1 suggest that economic forces and returns on investment do not provide adequate incentives for the private sector to address many of its measurement needs. Establishing consortia with industry, university, and government collaborators could be an effective way to address measurement needs and barriers that, if removed, will accelerate technological innovation. To be most effective in quickly bringing technological innovations to the marketplace, all members of a given MN consortium or team would have the same set of objectives, milestones, and outcomes for which they would be responsible and accountable.

“What will America do as a nation when Moore’s Law has beat(en) its last heartbeat; when it no longer delivers its productivity gains and anti-inflationary effects? How will we pay for ever-rising healthcare costs? What will happen if America’s economy falls behind and the U.S. is no longer the global leader? Other nations recognize the importance of semiconductors at the public level and are investing heavily. These are important questions for legislators to consider” [30].

## Figures and Tables

**Table 1:** Metrology Difficult Challenges Table - Adapted from the 2005 ITRS Table 116. The Summary of Issues column in the following Metrology Difficult Challenges Table that appeared in the 2005 ITRS as Table 116[2] lists where appropriate each of the 14 semiconductor case studies of measurement needs from Appendix B of Reference 1. The bold fonts designate the shorthand notations for these case studies. This Table was adapted from Table 116 with permission from the ITRS. Appendix 1 contains the page numbers for each of the case studies in Appendix B of Reference 1.

Difficult Challenges ≥32 nm	Summary of Issues
Factory level and company wide metrology integration for real-time in situ, integrated, and inline metrology tools; continued development of robust sensors and process controllers; and data management that allows integration of add-on sensors.	Standards for process controllers and data management must be agreed upon. Conversion of massive quantities of raw data to information useful for enhancing the yield of a semiconductor manufacturing process. Better sensors must be developed for trench etch end point, and ion species/energy/dosage (current). **Control**+
Starting materials metrology and manufacturing metrology are impacted by the introduction of new substrates such as SOI. Impurity detection (especially particles) at levels of interest for starting materials and reduced edge exclusion for metrology tools. CD, film thickness, and defect detection are impacted by thin SOI optical properties and charging by electron and ion beams.	Existing capabilities will not meet Roadmap specifications. Very small particles must be detected and properly sized. Capability for SOI wafers needs enhancement. Challenges come from the extra optical reflection in SOI and the surface quality. **Detection, X-ray, Microscopes**+
Control of high-aspect ratio technologies such as damascene challenges all metrology methods. Key requirements are dimensional control, void detection in copper lines, and pore size distribution and detection of killer pores in patterned low-k dielectrics.	New process control needs are not yet established. For example, 3D (CD and depth) measurements will be required for trench structures in new low-k dielectrics. Sidewall roughness impacts barrier integrity and the electrical properties of lines and vias. **Sidewall**+
Measurement of complex material stacks and interfacial properties including physical and electrical properties.	Reference materials and standard measurement methodology for new high-k gate and capacitor dielectrics with engineered thin films and interface layers as well as interconnect barrier and low-k dielectric layers, and other process needs. Optical measurement of gate and capacitor dielectric averages over too large an area and needs to characterize interfacial layers. Carrier mobility characterization will be needed for stacks with strained silicon and SOI substrates. The same is true for measurement of barrier layers. Metal gate work function characterization is another pressing need. **III–V Cluster Tools**+
Measurement test structures and reference materials.	The area available for test structures is being reduced especially in the scribe lines. There is a concern that measurements on test structures located in scribe lines do not correlate with in-die performance. Overlay and other test structures are sensitive to process variation, and test structure design must be improved to ensure correlation between measurements in the scribe line and on chip properties. Standards institutions need rapid access to state of the art development and manufacturing capability to fabricate relevant reference materials. **RF Isolation**+
Difficult Challenges ≥32 nm	
Nondestructive, production worthy wafer and mask-level microscopy for critical dimension measurement for 3D structures, overlay, defect detection, and analysis.	Surface charging and contamination interfere with electron beam imaging. CD measurements must account for sidewall shape. CD for damascene process may require measurement of trench structures. Process control such as focus exposure and etch bias will require greater precision and 3D capability. **SEM, III–V Cluster Tools**+
New strategy for in-die metrology must reflect across chip and across wafer variation.	Correlation of test structure variations with in-die properties is becoming more difficult as device shrinks. **Microscopes, RF Isolation, Electrical Properties**+
Statistical limits of sub-32 nm process control.	Controlling processes where the natural stochastic variation limits metrology will be difficult. Examples are low-dose implant, thin-gate dielectrics, and edge roughness of very small structures. **Nanoelectronics, Electrical Properties**+
Structural and elemental analysis at device dimensions and measurements for beyond CMOS.	Materials characterization and metrology methods are needed for control of interfacial layers, dopant positions, defects, and atomic concentrations relative to device dimensions. One example is 3D dopant profiling. Measurements for self-assembling processes are also required. **Distributions, Interfaces, 3D Mapping, Light Element Mapping**+
Determination of manufacturing metrology when device and interconnect technology remain unde-fined.	The replacement devices for the transistor and structure and materials replacement for copper interconnect are being researched. **Nanoelectronics, Spin, Electrical Properties**+

+ Please refer to Appendix 1 to locate the case study of the measurement need in Appendix B of Reference 1.

## 6. Appendix 1. Shorthand Notation Used in Table 1

This Appendix lists the page number and shorthand notation used in Table 1 for each of the 14 semiconductor case studies of measurement needs appearing in Appendix B of Reference 1.

**Table:**

Case Study of Measurement Need - Technology at Issue	Page Number in Appendix B of Reference 1	Shorthand Notation Used in Table 1
In-line Inspection and Factory Control Equipment	204	Control
In-line/Real-time Analytic Tools for Measuring and Detecting Sub-10 nm Defects	205	Detection
Dopant Distribution Instrumentation	206	Distributions
Interfacial Characterization Instrumentation	207	Interfaces
3D Atomic Mapping Instrumentation - Structural and Materials Properties	208	3D Mapping
Next-generation optical microscopes	209	Microscopes
Atomic Mapping Instrumentation - Light Elements	210	Light Element Mapping
Compound Semiconductor Cluster Tools	211	III–V Cluster Tools
Full System-on-Chip for Wireless Communications	212	RF Isolation
Sub-10 nm SEM Metrology Tools	213	SEM
Sidewall Characterization Instrumentation	214	Sidewall
Spin Metrology Tools	215	Spin
Semiconductor industry defect metrology tools	216	X-Ray
Instrumentation for Measurement of Electrical Properties at the Nanoscale	217	Electrical Properties
